# Polymeric Pseudo-Liquid Membranes from Poly(*N*-oleylacrylamide)

**DOI:** 10.3390/membranes4020210

**Published:** 2014-04-30

**Authors:** Hiroko Shiono, Masakazu Yoshikawa

**Affiliations:** Department of Biomolecular Engineering, Kyoto Institute of Technology, Matsugasaki, Kyoto 606-8585, Japan

**Keywords:** cesium ions, chiral separation, crown ether, liquid membrane, phenylglycine, polymeric pseudo-liquid membrane, poly(*N*-oleylacrylamide), potassium ions

## Abstract

A polymeric pseudo-liquid membrane (PPLM) was constructed from poly(*N*-oleylacrylamide) (PC18AAm), which exhibited a rubbery state under membrane transport conditions and used as the membrane matrix. In the present study, dibenzo-18-crown-6 (DB18C6) and dibenzo-21-crown-7 (DB21C7) were adopted as transporters for alkali metal ions. KCl was adopted as a model substrate for DB18C6 and CsCl the latter. Chiral transporter, *O*-allyl-*N*-(9-anthracenylmethyl)cinchonidinium bromide (AAMC) was used as a transporter for chiral separation of a racemic mixture of phenylglycine (Phegly). The l-somer was transported in preference to the antipode. The present study revealed that PPLMs are applicable to membrane transport, such as metal ion transport and chiral separation.

## 1. Introduction

Membrane separation is perceived to be an environmentally benign separation technology compared with other separation methods [1,2,3]. Membranes have gained much attention since they are expected to solve problems the globe is faced with that have to be immediately solved. Membranes have been already applied to many fields, such as production of drinking water from sea water by reverse osmosis (RO), production of ultrapure water by nanofiltration (NF), separation and concentration of macromolecules and colloidal particles by ultrafiltration (UF), removal of microorganisms by microfiltration (MF), concentration or removal of ionic materials by electrodialysis (ED), gas separation for recovery of H_2_, concentration of O_2_, and removal of CO_2_, dehydration or purification of biofuel by pervaporation (PV), hemofiltration, hemodiafiltration, hemodialysis, and so forth.

Systems for membrane separation can be divided into two categories, such as liquid membranes and solid membranes. The former directly and effectively reflects the affinity of the molecular recognition material (transporter or carrier), which is found in the liquid membrane. In addition to this, a construction of liquid membrane is easy, that is, dissolution of the transporter into a given solvent is just a manipulation that membranologists have to do. However, the drawback of a liquid membrane is its lack of long-term stability; the solvent consisting of membrane solution may evaporate, or the transporter and/or transporter/target molecule complex may be washed out during operation [4,5,6,7,8,9]. If these drawbacks are eliminated, a liquid membrane is a promising membrane system for the separation of target molecule from a mixture containing compounds with similar or same molecular dimensions and compounds that exhibit similar or the same chemical and/or physical properties.

There have been various approaches to endow liquid membranes with durability; (1) polymer liquid crystal composite membranes [10,11]; (2) polymer inclusion membranes [12,13,14,15,16,17,18,19,20,21,22]; (3) organogel membranes [23,24]; (4) stabilization of supported liquid membranes [25,26]; (5) room temperature ionic liquids [27,28]; and (6) polymeric pseudo-liquid membranes (PPLMs) [29,30,31,32,33,34,35]. PPLM is a liquid membrane that consists of polymeric materials in a rubbery state and a transporter for a given target molecule. In PPLMs, polymeric materials, which show rubbery state and fluidity, are chosen as membrane components dissolving a transporter and working as a barrier separating source and receiving phases. Exploration of more suitable membrane materials for PPLMs is not only an interesting but also an indispensable approach. From this, poly(2-ethylhexyl methacrylate) (P2EHMA) with glass transition temperature (*T*_g_) of −14.3 °C [32], poly(2-ethylhexyl acrylate) (P2EHA) with *T*_g_ of −60.5 °C [33], poly(dodecyl methacrylate) (PC12MA) with *T*_g_ of −66.3 °C [34], and poly(octadecyl methacrylate) (PC18MA) with *T*_g_ of −100 °C [35] were adopted as membrane matrices for PPLMs. Against expectation deduced from *T*_g_ values, PC18MA/DB18C6 PPLM did not give the highest normalized flux of K^+^ among above PPLMs. This might be due to the long alkyl moiety of PC18MA. The double bond leads to the introduction of a rigid 30° bend in the octadecyl chain. As a result, unsaturated octadecyl moieties, oleyl moieties, pack together less effectively than octadecyl ones. The reduced van der Waals interactions of oleyl moieties cause their melting points to decrease. Consequently, the fluidity of oleyl moieties would become more than that of PC18MA. To this end, in the present study, poly(*N*-oleylacryamide) (PC18AAm), of which *T*_g_ was reported to be −99 °C [36], was adopted as a candidate membrane matrix for a PPLM. PC18AAm is consisted of oleyl moiety, which has one double bond with *cis* configuration in the middle of it.

In the present study, the transport of KCl and CsCl through PPLMs from PC18AAm and dibenzo-18-crown-6 (DB18C6) for KCl or dibenzo-21-crown-7 (DB21C7) for CsCl and chiral separation of a recemic mixture of phenylglycine (Phegly) through a PC18AAm and *O*-allyl-*N*-(9-anthracenylmethyl)cinchonidium bromide (AAMC) membrane were investigated.

## 2. Results and Discussion

### 2.1. Preparation of PC18AAm

**Figure 1:**
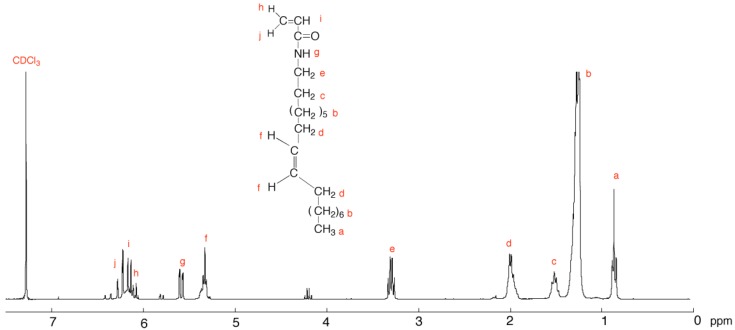
^1^H NMR spectrum of C18AAm (300 MHz, CDCl_3_).

Figure 1 shows the ^1^H NMR spectrum of *N*-oleylacryamide (C18AAm). The [M-Na^+^] value for C18AAm was determined to be 344.32, while the calculated value to be 344.60. ^1^H NMR and ESI-TOF MS spectra confirmed the successful synthesis of C18AAm.

In the present study, C18AAm was polymerized by radical polymerization using AIBN as a radical initiator. The number-average molecular weight of PC18AAm, *M*_n_, was determined to be 7.70 × 10^4^, and its polydispersity index, *M*_w_/*M*_n_, to be 1.52.

**Figure 2:**
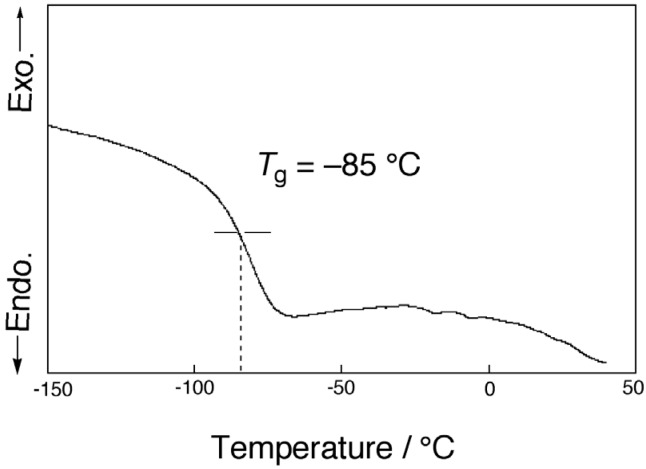
DSC thermograph of PC18AAm (Heating rate, 10 °C·min^−1^; N_2_, flow, 50 cm^3^·min^−1^).

Figure 2 shows the obtained differential scanning calorimetry thermograph of PC18AAm. The glass transition temperature, *T*_g_, of the present polymer was determined to be −85 °C, which was 14 °C higher than the reported value [36]. Contrary to PC18MA [35], the endothermic peak corresponding to the melting point was hardly observed at around 22 °C, where an endothermic peak for oleyl moieties was expected to be observed. The presence of the *cis*-double bond in the 9–10 position led to elimination of side-chain ordering. As a result, PC18AAm prepared in the present study showed the amorphous state.

### 2.2. Transport of KCl through the Membranes

**Figure 3:**
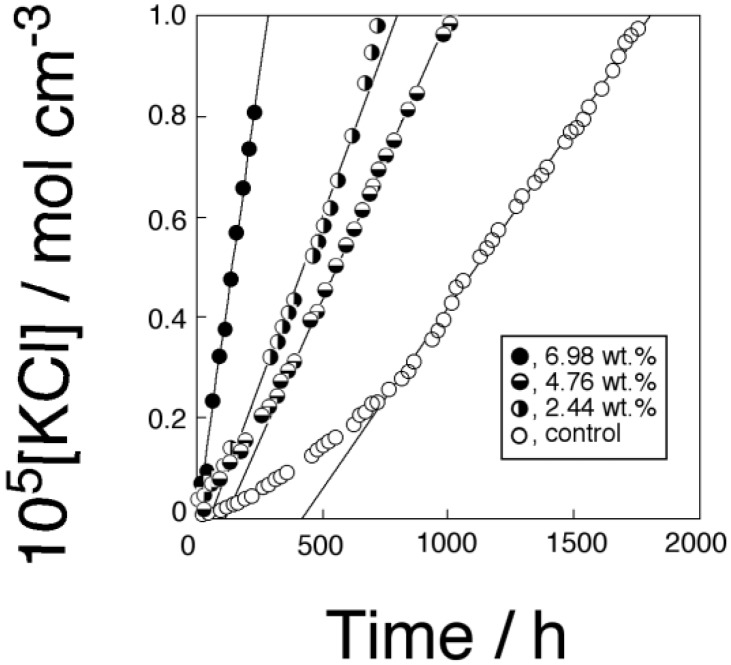
Time-transport curves of KCl through the PC18AAm liquid membranes (Operating temperature, 50 °C (323 K); [KCl]_0_ = 1.0 × 10^−4^ mol·cm^−3^).

DB18C6 was used as a model transporter and KCl was used as a target substrate to compare the present membrane with previous results [32,33,34,35]. Figure 3 shows the time-transport curves of KCl through the three types of PPLM that consist of PC18AAm and DB18C6 and through the corresponding control membrane. The straight line for each transport curve in Figure 3 was regarded as a steady state for each transport experiment. KCl was slightly transported through the control membrane, which consisted of just PC18AAm. The transport of KCl through the control membrane was thought to be a simple diffusion of KCl, the diffusion of free KCl and uncomplexed ion pairs through the membrane.

In the case of the transport of uni-univalent salt, such as KCl, in which the salt is transported through a given liquid membrane simultaneously by simple diffusion and by facilitated transport, the flux of the uni-univalent salt can be represented by the following Equation [37,38]:

*J*_C,obsd_ = (*D*_CA_*k*/δ)[K^+^]^2^ + (*D*_CLA_*kK*[DB18C6]/δ)[K^+^]^2^ (1)

**Figure 4:**
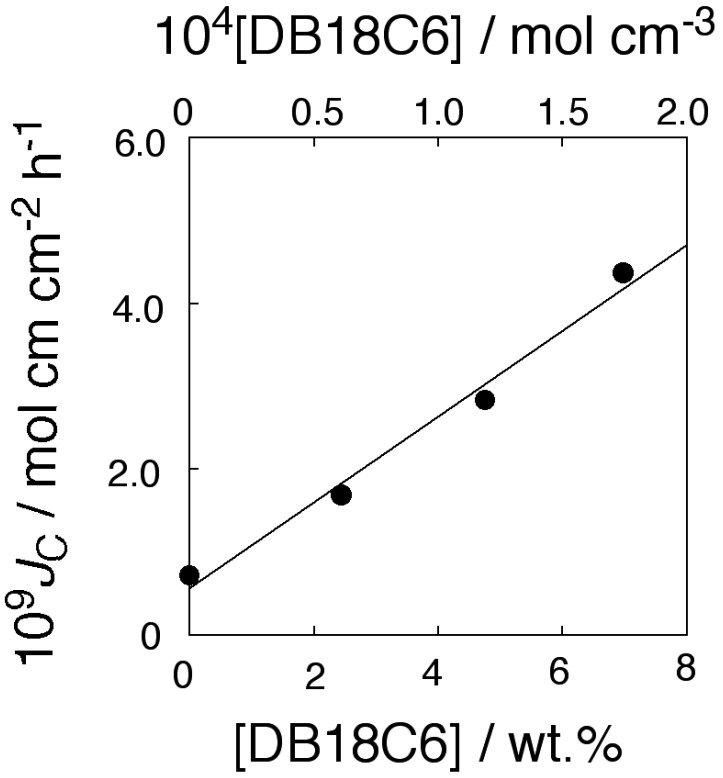
Relationship between KCl flux and the DB18C6 concentration through the PC18AAm liquid membranes (Operating temperature, 50 °C (323 K); [KCl]_0_ = 1.0 × 10^−4^ mol·cm^−3^).

Figure 4 shows the flux dependence on the transporter concentration. In Figure 4, the product of *J*_C,obsd_ and membrane thickness δ, *J*_C_ (mol·cm·cm^−2^·h^−1^), which is a flux per unit membrane thickness and per unit membrane area, is plotted as a function of transporter concentration. The total flux of K^+^ through the PPLM (*J*_C_) exhibited a linear relationship to the transporter concentration of DB18C6. The relationship held for the flux of control membrane, with no transporter. The relationship shown in Figure 4 revealed that the membrane transport of KCl was carried out by carrier-diffusion mechanism [37,38] not by fixed-site jumping [15,39,40]. This revealed that the membrane matrix of PC18AAm was fluid enough so that transporter and transporter/substrate complex could diffuse freely within the membrane. In the present study, KCl was transported like usual liquid membrane [37,38].

**Figure 5:**
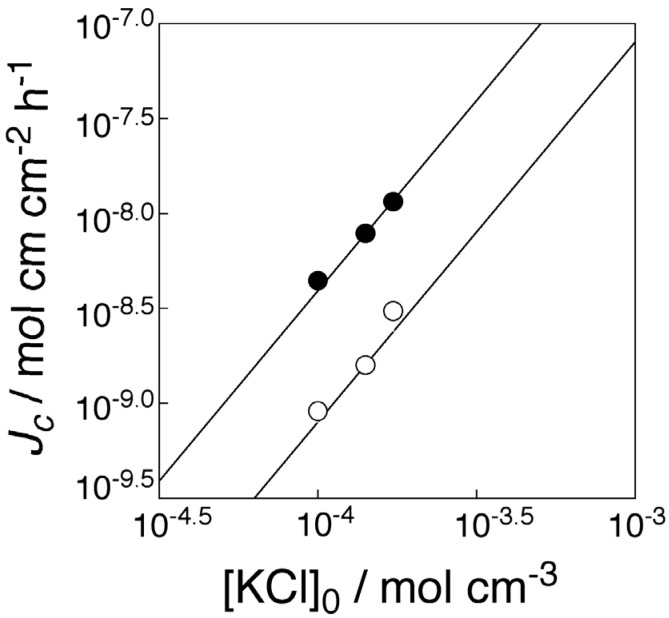
Dependence of facilitated KCl transport and simple diffusion fluxes on KCl feed concentration at the operating temperature of 50 °C (323 K) ([DB18C6] = 1.32 × 10^−4^ mol·cm^−3^).

Following Equation (1), both K^+^ flux facilitated by transporter and that of simple diffusion should be dependent on the square of the initial feed K^+^ concentration. To confirm this, the logarithms of both K^+^ fluxes are plotted as functions of the logarithms of the initial K^+^ concentrations (Figure 5). Figure 4 and Figure 5 revealed that K^+^ transport through the present membranes took place by means of the mobile carrier mechanism.

### 2.3. Dependence of Membrane Transport on Operating Temperature

**Figure 6:**
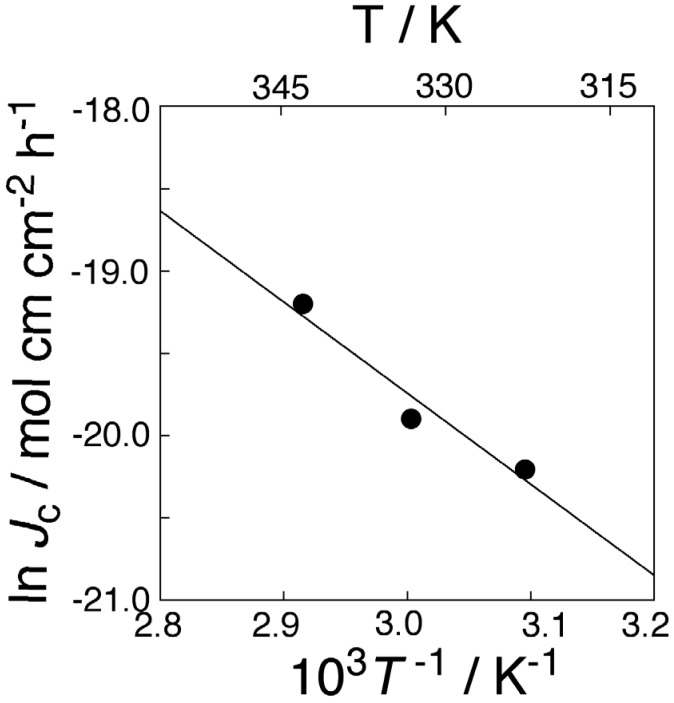
Temperature dependence of KCl transport through the PC18AAm liquid membrane ([DB18C6] = 1.32 × 10^−4^ mol·cm^−3^; [KCl]_0_ = 1.0 × 10^−4^ mol·cm^−3^).

The membrane transport of K^+^ through the present PPLM, of which DB18C6 concentration of 1.32 × 10^−4^ mol·cm^−3^ (4.76 wt %), was studied at three different operating temperatures, such as 70 °C (343 K), 60 °C (333 K), and 50 °C (323 K). Adopting flux values for those three types of membrane transport experiment, Arrhenius plot of K^+^ flux was plotted against reciprocal of absolute temperature, which is shown in Figure 6. From the slope of the straight line in the figure, the activation energy of K^+^ transport was determined to be 45.7 kJ·mol^−1^.

**Figure 7:**
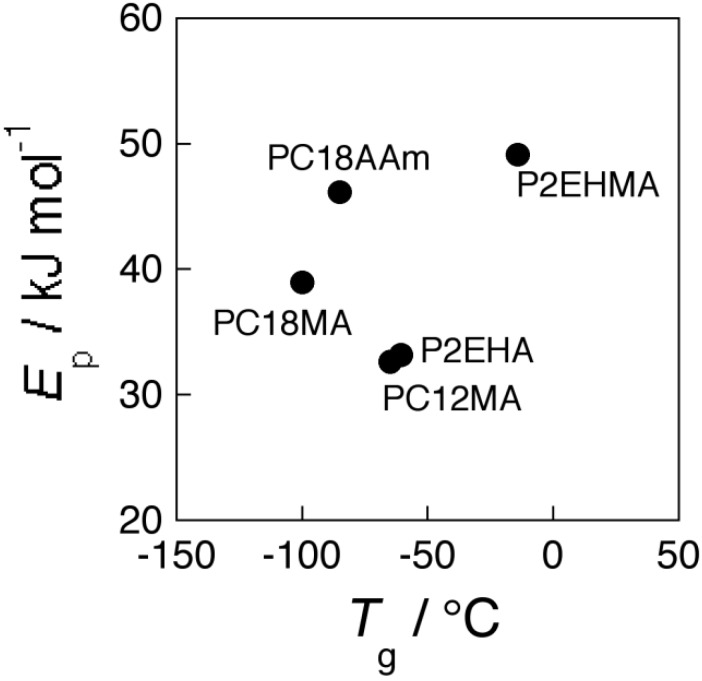
Relationship between activation energy of KCl transport and glass transition temperature of membrane matrix.

So far, four types of polymeric materials were studied as membrane matrices for PPLM. The relationship between activation energy of membrane transport of K^+^ and the glass transition temperature is shown in Figure 7. As anticipated from previous study [35], the present membrane from PC18AAm gave higher activation energy than those for P2EHMA and PC12MA. However, the activation energy of PC18AAm of 45.7 kJ·mol^−1^ was an expected value among PPLMs consisting of side chain with 18 carbons, since PC18AAm showed higher *T*_g_ value than PC18MA. From Figure 7, the plots of P2EHMA, P2EHA, and PC12MA seem to constitute one group and those of PC18AAm and PC18MA to form another one. The former group and the latter one seem to form each straight line independently. Those two straight lines would cross at around −260 °C (13 K). Accumulation of the relationship between activation energy of membrane transport and the corresponding *T*_g_ value would lead to an interesting result.

### 2.4. Comparison of the Present PPLM with Others

The flux values observed in the present study are summarized in Table 1 together with results for PPLMs from P2EHMA [32], P2EHA [33], PC12MA [34], and PC18MA [35], supported liquid membrane [41], and polymer inclusion membrane [14]. In the table, normalized fluxes, which are fluxes per unit membrane thickness, per unit membrane area, per unit transporter concentration, and per square of unit substrate concentration, are given so that each membrane performance can be compared. In the case of polymer inclusion membrane, the result obtained, using dicyclohexyl-18-crown-6 (DC18C6) as a transporter instead of DB18C6, was cited.

**Table 1:** Comparison of normalized K^+^ flux values for various membranes.

Liquid membrane	*J* (normalized flux of K^+^)	Flux ratio ^a^	Operating temperature (°C)
	(mol·cm·cm^−2^·h^−1^)		
	(mol·cm^−3^)(mol·cm^−3^)^2^		
PC18AAm/DB18C6 ^b^	6.75 × 10^3^	40	70
PC18AAm/DB18C6 ^b^	6.43 × 10^3^	38	60
PC18AAm/DB18C6 ^b^	4.86 × 10^3^	29	50
PC18MA/DB18C6 ^c^	6.89 × 10^3^	41	60
PC12MA/DB18C6 ^d^	5.79 × 10^4^	350	40
P2EHA/DB18C6 ^e^	5.88 × 10^3^	35	40
P2EHMA/DB18C6 ^f^	6.20 × 10^3^	37	40
CHCl_3_/DB18C6 ^g^	1.67 × 10^2^	1	25
PIM/DC18C6 ^h^	2.37 × 10^2^	1.4	25

^a^ Flux ratios are the relative values; the flux value for the supported liquid membrane being set as unity; ^b^ Present study; ^c^ Cited from [35]; ^d^ Cited from [34]; ^e^ Cited from [33]; ^f^ Cited from [32]; ^g^ Cited from [41]; ^h^ Cited from [14].

PC12MA still showed the highest normalized flux value among those PPLMs. The long alkyl moiety might reduce the normalized flux of PC18AAm against expectation deduced from *T*_g_ values. Adopting PC18AAm with lower molecular weight as membrane matrix, a better membrane performance would be observed as observed in the study on P2EHA [33].

### 2.5. Transport of CsCl through the Membrane

As expected from the composition of liquid membrane, the membrane performance is exclusively dependent on the nature of transporter in the membrane. The situation is the same for PPLM. From this, dibenzo-21-crown-7 (DB21C7) [42,43] was adopted as a transporter instead of DB18C6, membrane transport of Cs^+^ was studied since the isotope ^135^Cs, which is formed in nuclear reactors, has a long half-life of 2.3 × 10^6^ years, ^137^Cs 30.17 years, ^134^Cs 2 years, and so forth [44,45].

**Figure 8:**
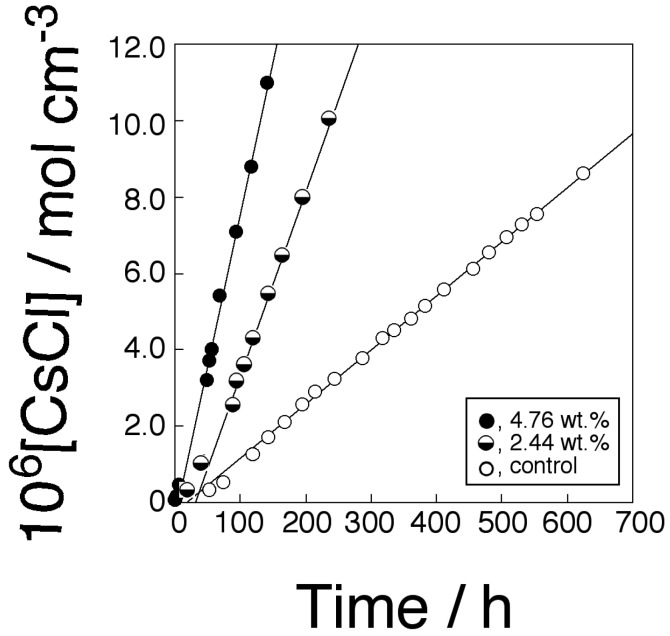
Time-transport curves of CsCl through the PC18AAm liquid membrane (Operating temperature, 50 °C (323 K); [CsCl]_0_ = 1.0 × 10^−4^ mol·cm^−3^).

**Figure 9:**
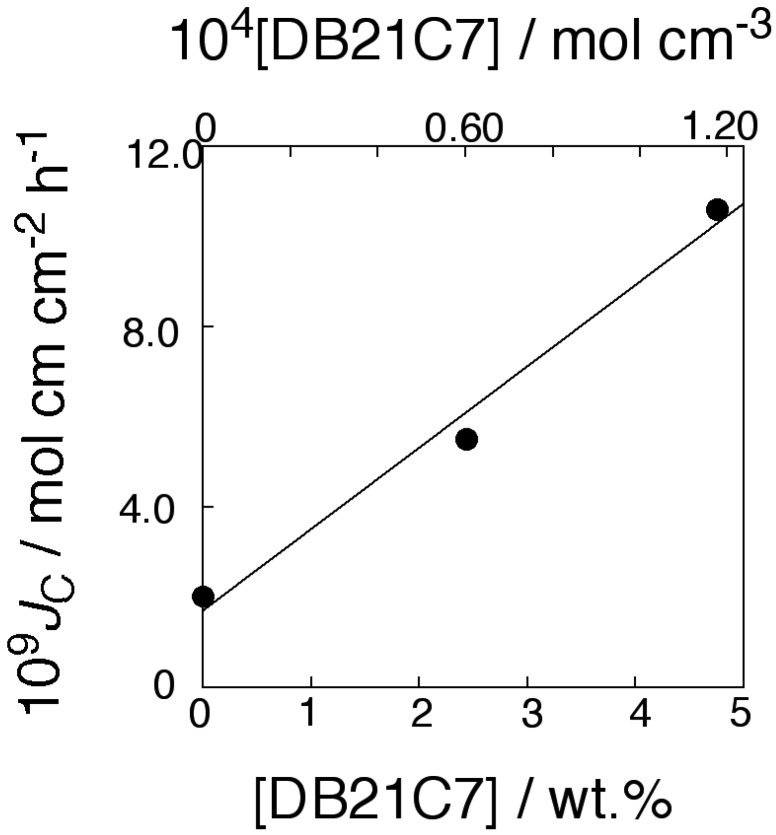
Relationship between CsCl flux and the DB21C7 concentration through the PC18AAm liquid membrane (Operating temperature, 50 °C (323 K); [CsCl]_0_ = 1.0 × 10^−4^ mol·cm^−3^).

Figure 8 shows time-transport curves of CsCl through PPLMs that consisted of PC18AAm and DB21C7 and the corresponding control membrane. The flux dependence on transporter concentration is shown in Figure 9. Figure 9 revealed that the transport of CsCl was also carried out by the mobile carrier mechanism like that of KCl through the present PC18AAm/DB18C6 membrane.

### 2.6. Chiral Separation of Racemic Mixture of Phenylglycine (Phegly)

In the previous sections, it was confirmed that the membrane performance of liquid membrane is greatly dependent on the transporter in it and that the membrane matrix consisting of a given liquid membrane can be transformed to be any membranes to separate any target materials. In the previous studies, the authors confirmed that PPLMs had potential to resolve enantiomers [34,35]. Separation of chiral compounds is an interesting and important subject since optical resolution is carried out in industries such as pharmaceuticals, agrochemicals, food additives, perfumes, and so forth [46,47,48,49,50].

*O*-Allyl-*N*-(9-anthracenylmethyl)cinchonidinium bromide (AAMC) was adopted as a transporter showing chiral recognition ability since cinchona alkaloids have been used as resolving agents for chiral binaphthols [51], chiral acids [52], amino acid derivatives [52,53], and oligopeptides [54]. Cinchona alkaloid was also used as a transporter for optical resolution [55]. Based on those studies described above, AAMC was chosen as a transporter for enantioselective transport. A racemic mixture of phenylglycine (Phegly) was adopted as a model racemate.

**Figure 10:**
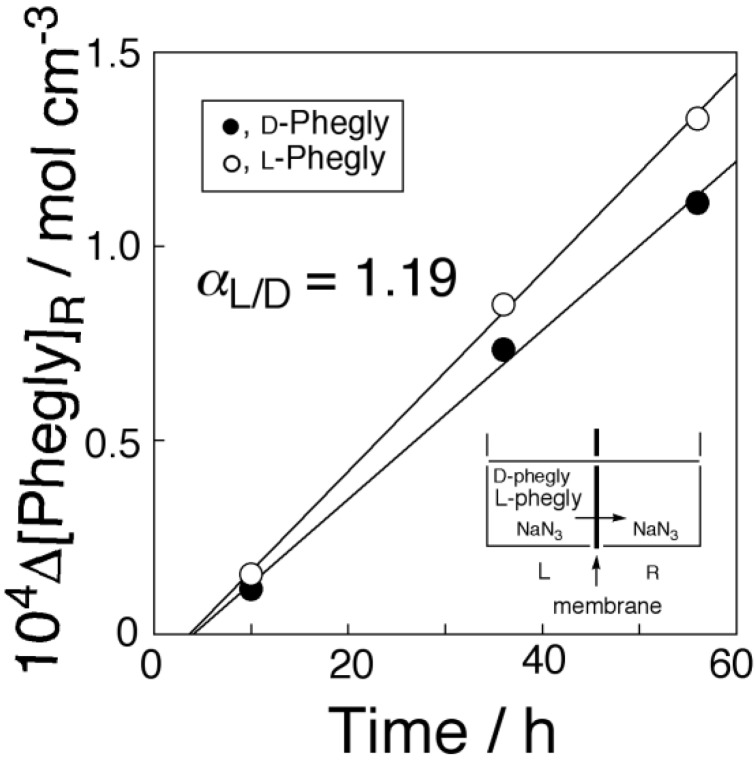
Chiral separation of reacmic mixture of Phegly through the PC18AAm liquid membrane (Operating temperature, 50 °C (323 K); [AAMC] = 4.13 × 10^−5^ mol·cm^−3^: [d-Phegly]_L,0_ = [l-Phegly]_L,0_ = 1.0 × 10^−6^ mol·cm^−3^).

Figure 10 shows the time-transport curves of racemic mixture of Phegly through the membrane. The l-isomer of Phegly was preferentially transported through the membrane as expected [34,35,55]. The permselectivity toward l-Phegly was determined to be 1.19. The liquid membrane [55] and PPLMs [34,35] bearing AAMC as a transporter gave higher permselectivity than that observed in the present study. The membrane performance for chiral separation was dependent on experimental conditions. It was already reported that the permselectivity was controlled from l-isomer permselectivity to d-isomer one by adjusting the operating temperature [35]. The membrane performance of the present membrane would be improved by adjusting operating conditions.

## 3. Experimental Section

### 3.1. Materials

**Figure 11:**
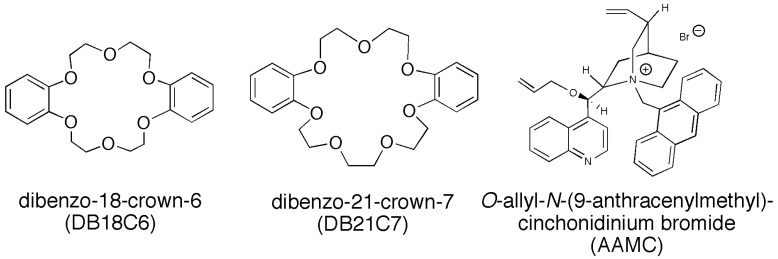
Chemical structures of various transporters.

Acryloyl chloride, *N*-oleylamine, toluene, chloroform, KCl, CsCl, d-phenylglycine (d-Phegly), and l-phenylglycine (l-Phegly) were purchased from Nacalai Tesque, Inc. (Kyoto Japan) and used without further purification. Dibenzo-18-crown-6 (DB18C6), dibenzo-21-crown-7 (DB21C7), and *O*-allyl-*N*-(9-anthracenylmethyl)cinchonidinium bromide (AAMC) (Figure 11) were obtained from Sigma-Aldrich (St. Louis, MO, USA) and used as received. *N*-oleylacrylamide (C18AAm) was prepared from acryloyl chloride and *N*-oleylamine [56]. 2,2′-Azobis(2-methylpropionitrile) (AIBN) [57] and toluene [58] were purchased from Nacalai Tesque, Inc. (Kyoto, Japan) and purified by conventional methods. Water purified with an ultrapure water system (Simpli Lab, Millipores S.A., Molsheim, France) was used.

### 3.2. Characterization of C18AAm

The ^1^H NMR spectrum was recorded in CDCl_3_ using a BRUKER AV-300 (Bruker Co., Billerica, MA, USA) with residual partially protonated solvent of CDCl_3_ as an internal standard (δ = 7.26 ppm [59]).

Mass spectrum of C18AAm was recorded using a BRUKER microTOF LC (Bruker Co., Billerica, MA, USA).

### 3.3. Preparation of poly(N-oleylacrylamide) (PC18AAm)

**Figure 12:**
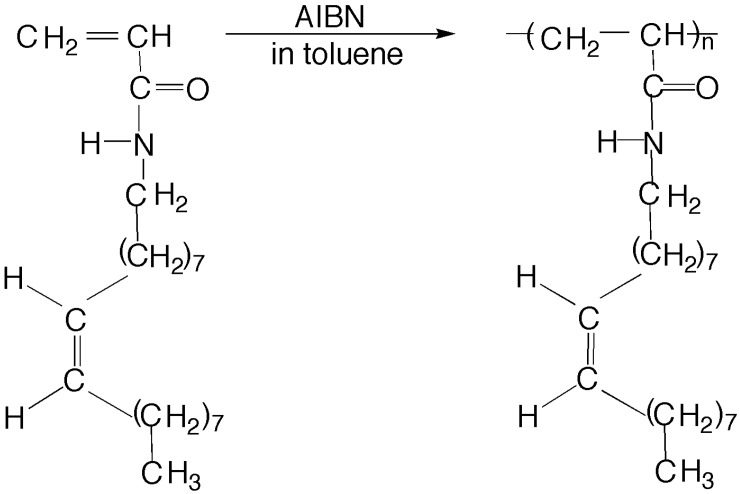
Preparation of PC18AAm.

The polymerization scheme was shown in Figure 12. PC18AAm was synthesized as follows: 3.86 g (1.2 × 10^−2^ mol) of C18AAm, 10.2 mg (6.3 × 10^−5^ mol) of AIBN, and 46 cm^3^ of toluene were placed in an ampoule. The ampoule containing above mixture was degassed three freeze-pump-thaw cycles and sealed off under reduced pressure, which was below 1.3 × 10^−2^ Pa (1.0 × 10^−4^ mmHg). The sealed ampoule was shaken in a water bath at a constant temperature of 45 °C for 144 h. The solution was poured into methanol, which was kept around −80 °C and the resulting precipitate was collected. The polymer PC18AAm thus obtained was dried *in vacuo*. A 2.69 g (yield, 69.7%) of PC18AAm was obtained.

### 3.4. Characterization of PC18AAm

Gel permeation chromatograph (GPC) was performed on a JASCO liquid chromatography system composed of a PU-2089 HPLC pump (JASCO Co., Hachioji, Japan) and an 890-CO column oven (JASCO Co., Hachioji, Japan) (operated at 35 °C) equipped with a JASCO 870-UV (JASCO Co., Hachioji, Japan) and a Shodex RI-101 detector (Showa Denko K.K., Tokyo, Japan). Polystyrene standards (Tosoh Co., Tokyo, Japan) were used for calibration and THF as eluent at a flow rate of 1.0 cm^3^·min^−1^.

Differential scanning calorimetry (DSC) measurements were performed with Shimadzu DSC-60 (Shimadzu Co., Kyoto, Japan). The heating rate was fixed to be 10 °C·min^−1^ and the sample was purged with nitrogen at a flow rate of 50 cm^3^·min^−1^.

### 3.5. Preparation of Polymeric Pseudo-Liquid Membranes

Polymeric pseudo-liquid membrane was prepared as follows: about 100.0 mg of PC18AAm and the prescribed amount of DB18C6, of which amount was 2.50 mg, 5.00 mg, and 7.50 mg, were dissolve in 1.0 cm^3^ of CHCl_3_. In the case that DB21C7 was used as a transporter instead of DB18C6, about 100.0 mg of PC18AAm and 2.50 mg of DB21C7 or 5.00 mg of that were dissolved in 1.0 cm^3^ of CHCl_3_. The polymer solution was poured into a flat-laboratory-dish (48 mm diameter), followed by immersing a PTFE membrane filter (Omnipore Membrane Filter (Merck Millipore, Bellerica, MA, USA); diameter 47 mm; pore radius, 0.10 µm; porosity 0.80; thickness, 80 µm) into the cast solution. Then, the flat-laboratory-dish was evacuated in a desiccator so that the cast solution could thoroughly penetrate into pores in the PTFE membrane filter. The solvent was allowed to evaporate at 25 °C for 5 h and then additionally at 60 °C for 24 h.

Control membrane was prepared as follows: 100.0 mg of PC18AAm was dissolved in 1.0 cm^3^ of CHCl_3_. Control membrane for PPLM was constructed from the solution thus prepared as described above.

PPLM for chiral separation of Phegly was prepared as described above. Instead of transporter for alkali metal ion, 2.50 mg of *O*-allyl-*N*-(-9-anthracenylmethyl)cinchonidinium bromide (AAMC) was adopted as a transporter for chiral separation of racemic mixture of Phegly.

### 3.6. Transport of Alkali Metal Salts

**Figure 13:**
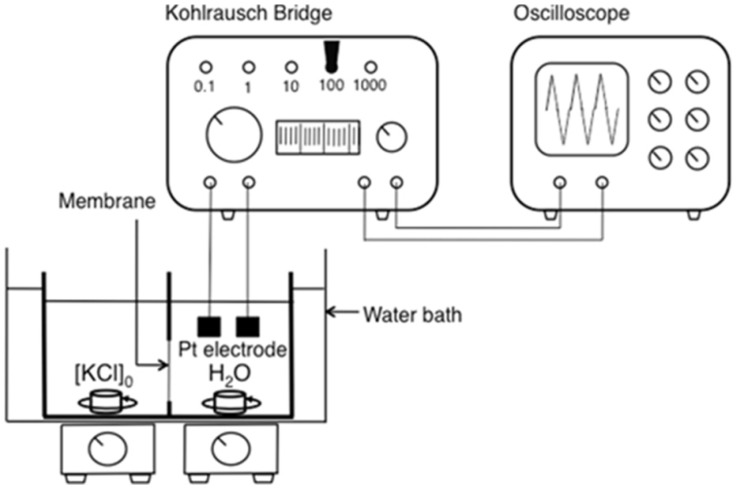
Schematic representation of the setup for alkali metal ion transport.

Transport of alkali metal salt, such as KCl and CsCl, was studied using apparatus schematically shown in Figure 13. The PTFE filter membrane impregnated with membrane component, such as PC18AAm and transporter or just PC18AAm, was secured tightly with Parafilm between two chambers of a permeation cell. The thickness of the PTFE filter, 80 µm, was adopted as a membrane thickness for the present study. In the present study, the membrane area for PTFE filter membrane was 3.0 cm^2^; the effective membrane area was determined to be 2.4 cm^2^. The volume of each chamber was 40.0 cm^3^. A 1.0 × 10^−4^ mol·cm^−3^ of KCl or CsCl aqueous solution was placed in the left-hand side chamber (L-side) and deionized water in the right-hand side chamber (R-side). Transport experiments were carried out at 70 °C (343 K), 60 °C (333 K), and 50 °C (323 K) for KCl transport, and at 50 °C (323 K) for CsCl transport, respectively. Aqueous solutions in both chambers were stirred by magnetic stirrers. The revolution rate of magnetic stirrer was kept apparently constant as possible, though that could not be specified in the present study. Concentration of KCl or CsCl in the permeate side (R-side) was determined by conductometric analysis by using Portable Kohlrausch Bridge TYPR BF-62A (Shimadzu Rika Instruments Co., Ltd., Kyoto, Japan) and CO-1305 oscilloscope (Kenwood Co., Hachioji, Tokyo), of which a schematic diagram is shown in Figure 13.

### 3.7. Transport of Racemic Mixture of Phegly

Aqueous solution of racemic Phegly was placed in the left-hand side chamber (L-side) and aqueous solution in the right-hand side chamber (R-side). Each concentration of racemic Phegly was fixed to be 1.0 × 10^−6^ mol cm^−3^. Transport experiment was carried out at 50 °C (323 K). The pH condition of the source phase (L-side) was kept to be 11 by Na_2_HPO_4_/NaOH and that of the receiving phase was maintained at pH 3 by H_3_PO_4_/NaH_2_PO_5_.

The amounts of D-Phegly and L-Phegyl transported through the PPLM were determined by liquid chromatography (LC) [JASCO PU-2080, equipped with a UV detector (JASCO UV-2075) (JASCO Co., Hachioji, Japan)], using a CHIRALPAK MA(+) column [50 × 4.6 mm (i.d.)] (Daicel Co., Osaka, Japan) and aqueous copper sulfate as an eluent.

The permselectivity α_L/D_ is defined as the flux ratio, *J*_L_/*J*_D_, divided by the concentration ratio [l-Phegly]/[d-Phegly]

α_L/D_ = (*J*_L_/*J*_D_)/([L-Phegly]/[D-Phegly]) (2)

## 4. Conclusions

A polymeric pseudo-liquid membrane (PPLM) was constructed from poly(*N*-oleylacrylamide) (PC18AAm), which exhibited a rubbery state under membrane transport conditions and used as the membrane matrix. In the present study, dibenzo-18-crown-6 (DB18C6) and dibenzo-21-crown-7 (DB21C7) were adopted as transporters for alkali metal ions. KCl was adopted as a model substrate for DB18C6 and CsCl the latter. Chiral transporter, *O*-allyl-*N*-(9-anthracenylmethyl)cinchonidinium bromide (AAMC) was used as a transporter for chiral separation of a racemic mixture of phenylglycine (Phegly). The l-isomer was transported in preference to the antipode. The present study revealed that PPLMs are applicable to membrane transport, such as metal ion transport and chiral separation.

## Nomenclature

*D*_CA_: diffusion coefficient of the free solute (cm^2^·h^−1^)
*D*_CLA_: diffusion coefficient of the complexed solute (cm^2^·h^−1^)
*J*_C_: total flux of the diffusing solute, K^+^, across the membrane per unit membrane thickness (*J*_C_ = δ*x **J*_C,obsd_) (mol·cm·cm^−2^·h^−1^)
*J*_C,obsd _**: observed total flux of the diffusing solute, K^+^, across the membrane (mol·cm^−2^·h^−1^)
*k*: partition coefficient of the solute between water and the organic membrane
*K*: equilibrium constant for the association (mol^−1^·cm^3^)
δ: membrane thickness (cm)
[DB18C6]: total concentration of complexed and uncomplexed transporter, DB18C6, in the membrane (mol·cm^−3^)
[K^+^]: concentration of the diffusing solutes, K^+^, in the source phase (mol·cm^−3^)
